# Low anti-Müllerian hormone levels are associated with an increased risk of incident early-onset vasomotor symptoms among premenopausal women

**DOI:** 10.1038/s41598-022-16182-7

**Published:** 2022-07-13

**Authors:** SunJu NamGoung, Yoosoo Chang, Yejin Kim, Hoon Kim, In Young Cho, Ria Kwon, Ga-Young Lim, Hye Rin Choi, Jeonggyu Kang, Kye-Hyun Kim, Yun Soo Hong, Di Zhao, Hyun-Young Park, Juhee Cho, Eliseo Guallar, Min-Jung Kwon, Seungho Ryu

**Affiliations:** 1 Center for Cohort Studies, Total Healthcare Center, Kangbuk Samsung Hospital, Sungkyunkwan University School of Medicine, Seoul, Republic of Korea; 2 Department of The Environmental Health Center, Wonju Severance Christian Hospital, Yonsei University School of Medicine, Wonju, Republic of Korea; 3 Department of Occupational and Environmental Medicine, Kangbuk Samsung Hospital, Sungkyunkwan University School of Medicine, Samsung Main Building B2, 250, Taepyung-ro 2ga, Jung-gu, Seoul, 04514 Republic of Korea; 4 Department of Obstetrics and Gynecology, Seoul National University College of Medicine, Seoul, Republic of Korea; 5 Department of Family Medicine, Kangbuk Samsung Hospital, Sungkyunkwan University School of Medicine, Seoul, Republic of Korea; 6 Institute of Medical Research, Sungkyunkwan University School of Medicine, Suwon, Republic of Korea; 7 Department of Obstetrics and Gynecology, Kangbuk Samsung Hospital, Sungkyunkwan University School of Medicine, Seoul, Republic of Korea; 8 Departments of Epidemiology and Medicine, and Welch Center for Prevention, Epidemiology, and Clinical Research, Johns Hopkins University Bloomberg School of Public Health, Baltimore, MD USA; 9 Department of Precision Medicine, National Institute of Health, Korea Disease Control and Prevention Agency (KDCA), Cheongju, Republic of Korea; 10 Department of Clinical Research Design and Evaluation, SAIHST, Sungkyunkwan University, Seoul, Republic of Korea; 11 Department of Laboratory Medicine, Kangbuk Samsung Hospital, Sungkyunkwan University School of Medicine, Saemunan-ro, Jongno-gu, Seoul, 03181 Republic of Korea

**Keywords:** Endocrinology, Signs and symptoms

## Abstract

The role of anti-Müllerian hormone (AMH) levels in incident vasomotor symptoms (VMS) is largely unknown. This study aimed to investigate the relationship between AMH levels and the development of early-onset VMS among premenopausal women. Our cohort study comprised 2041 premenopausal women aged 42–52 years free of VMS at baseline whose AMH levels were measured. VMS, including hot flushes and night sweats, were assessed using the Korean version of the Menopause-specific Quality of Life questionnaire. Early-onset VMS was defined as the occurrence of VMS prior to menopause. Parametric proportional hazards models were used to estimate adjusted hazard ratios (HRs) and 95% CI. During a median follow-up of 4.4 years, 708 premenopausal women developed early-onset VMS (incidence rate, 8.0 per 100 person-years). Lower AMH levels were statistically significantly associated with an increased risk of early-onset VMS. After adjusting for age and other confounders, multivariable-adjusted HRs (95% CI) for incident VMS comparing AMH quintiles 4–1 to the highest quintile were 1.02 (0.78–1.33), 1.37 (1.06–1.76), 1.36 (1.04–1.76), and 2.38 (1.84–3.08), respectively (*P* for trend < 0.001). Our results support an independent role of serum AMH levels in predicting incident early-onset VMS among premenopausal women in the late reproductive stage.

## Introduction

Vasomotor symptoms (VMS), including hot flushes and night sweats, are cardinal climacteric symptoms that negatively affect the quality of life of women^1,2^. VMS were previously considered to occur near the final menstrual period and last for a short duration, ranging from 6 months to 2 years. However, recent reports indicate that VMS are commonly observed in the premenopausal and early menopausal transition stages and may persist long after menopause^3–5^. Moreover, early-onset and long-lasting VMS were related to adverse psychosocial and physical outcomes, including an increased risk of subclinical and cardiovascular disease^6–8^. Additionally, while the mechanisms underlying early-onset and late-onset VMS^8^ may be different, only limited information on the differences in risk factors for early-onset and late-onset VMS is available^3^.

Early-onset VMS is considered a physiological response to hormonal fluctuations during menopausal transition^8,9^. However, the relationship between reproductive hormones and VMS is not completely understood. A large number of studies have focused on the role of estrogens in VMS because of their role in thermoregulation^10–12^, but estrogen decline alone does not fully explain the occurrence of VMS^9,13^. Anti-Müllerian hormone (AMH) has emerged as a reliable marker of ovarian function and a predictor of the time to menopause^14–16^. AMH levels can predict the time of the final menstrual period among late reproductive-age women, and are even better predictive indicators than follicle-stimulating hormones (FSH)^15,16^. Given the close association between AMH levels and the likelihood of experiencing menopause, it is hypothesized that AMH levels may predict VMS risk during the menopausal transition. Until now, studies addressing the effect of AMH on VMS risk have been limited. A cross-sectional study showed an association between AMH and hot flashes^13^. However, no cohort study has explored the association between AMH and early-onset VMS.

Therefore, this longitudinal study aimed to investigate the association between AMH levels and the risk of incident VMS among premenopausal women.

## Materials and methods

### Study population

**Figure 1:**
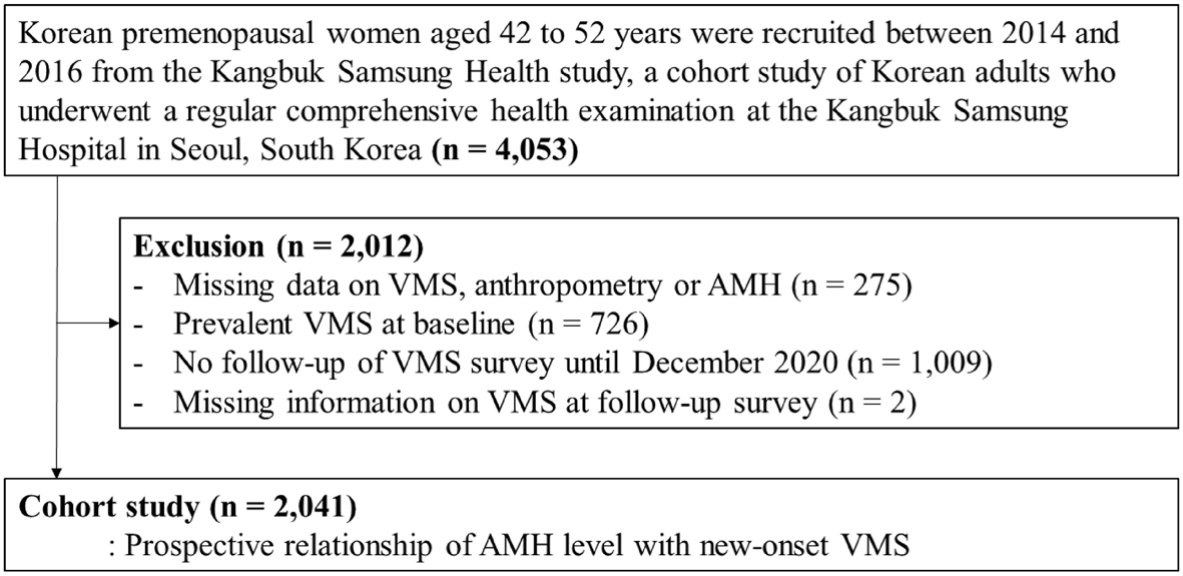
Flowchart of study participants.

For a longitudinal study of midlife Korean women, participants were recruited between 2014 and 2018 from the Kanbuk Samsung Health Study, a cohort study of Korean adults who received annual or biennial comprehensive health examinations at the clinics of Kangbuk Samsung Hospital Total Healthcare Centers in Seoul and Suwon, South Korea. The eligibility criteria for enrollment included (1) age 42–52 years; (2) no history of hysterectomy, oophorectomy, or hormone replacement therapy; (3) at least one menstrual period in the 3 months prior to the health screening exam and no amenorrhea lasting for ≥ 60 days; and (4) no history of a chronic disease that may affect menstrual cycles (malignancy, renal failure, and hypo- or hyperthyroidism). Of participants initially enrolled, we restricted to the premenopausal stage women from 2014 to 2016 at baseline when AMH was measured (n = 4053); and then we excluded 275 women with missing information on VMS, anthropometry or AMH level, 726 women who reported VMA at baseline, 1009 women with no follow-up visit, and 2 women with missing information on VMS. The final sample for the cohort study included 2,041 VMS-free premopausal women (Fig. 1).

### Measurements

AMH levels were measured at only baseline while VMS, menopausal stage, and other covariates were measured at baseline and follow-up visits. Information regarding demographic characteristics, lifestyle factors, reproductive factors, and medication use was obtained through a standardized, structured, self-administered questionnaire. Participants who had smoked < 100 cigarettes in their lifetime were classified as never smokers. Ever-smokers were defined as those who smoked ≥ 100 cigarettes in their lifetime (former and current smokers were not separated because of the very low prevalence of smoking). Average alcohol intake was categorized as none, ≤ 10 g/day, > 10 g/day, or unknown^9^. Physical activity was assessed using the validated Korean version of the International Physical Activity Questionnaire short form and categorized as inactive, minimally active, and health-enhancing physical activity^17^. Education level was categorized as less than college graduate versus college graduate or higher. Parity, as a reproductive factor, was defined as the number of previous pregnancies, including live births and stillbirths.

Menopausal stages were categorized based on the criteria of the Stages of Reproductive Aging Workshop + 10 as (1) Premenopause; (2) Early menopausal transition, defined as a persistent difference of ≥ 7 days in the length of consecutive cycles; (3) Late menopausal transition, defined as amenorrhea of ≥ 60 days; (4) Postmenopause, defined as amenorrhea of ≥ 12 months^18,19^.

Height and weight were measured by trained nurses, with the participants wearing a lightweight hospital gown and no shoes. Body mass index (BMI) was calculated as weight in kilograms divided by height in meters squared. BMI was categorized according to Asian-specific criteria as underweight (BMI < 18.5 kg/m^2^), normal weight (BMI 18.5–22.9 kg/m^2^), overweight (BMI 23–24.9 kg/m^2^) and obesity (BMI ≥ 25 kg/m^2^)^20^. Hypertension was defined as a systolic blood pressure (BP) ≥ 130 mmHg, diastolic BP ≥ 80 mmHg^21^, or current use of antihypertensive medication.

Blood samples were taken from the antecubital vein after at least a 10-h fast and were assayed for glucose, glycated hemoglobin, and AMH. Diabetes mellitus was defined as a fasting serum glucose ≥ 126 mg/dL, glycated hemoglobin ≥ 6.5% (48 mmol/mol), or current use of insulin or glucose-lowering medication.

AMH was measured using an Elecsys AMH Plus instrument on the Cobas e801 immunoassay analyzer (Roche Diagnostics, Tokyo, Japan). The analytical performance for precision of the Elecsys AMH assay was evaluated according to CLSI-EP15-A2 guidelines^22^; the inter-assay coefficients of variation for quality control specimens of lower levels and higher levels for AMH were 1.79–3.87% and 0.94–3.85%, respectively, during the study period. The limit of detection was determined in accordance with the CLSI EP17-A2 guidelines^23^ and reported as < 0.01 ng/mL. Blood sampling was performed regardless of the menstrual cycle phase, but AMH remains constant during the menstrual cycle^24^.

### Prevalent and incident VMS

At baseline and each follow-up visit, we used the Korean version of the Menopause-Specific Quality of Life questionnaire (MENQoL)^25,26^ to assess the presence and bothersome degree of VMS, including hot flushes and night sweats. If the participant responded “No” to hot flushes or night sweats, she was considered not to have VMS. If the participant responded “Yes” and experienced hot flushes or night sweats and rated them on the “bothered” scale, she was considered to have VMS.

### Statistical analyses

Currently, there are no established cutoff points for AMH to predict VMS or menopausal symptoms. To assess the relationship between the AMH levels as a continuous variable and the development of VMS, we modeled the AMH levels as restricted cubic splines with knots at the 5th, 27.5th, 50th, 72.5th, and 95th percentiles of the sample distribution to provide a flexible estimate of the concentration–response relationship between the AMH levels and incident VMS. Then, defined quintiles of AMH levels within the study population as follows: 0.01–0.199, 0.2–0.488, 0.489–0.894, 0.895–1.64 and 1.65–30.99 ng/mL. The fifth quintile representing the highest AMH level was used as the reference group.

The characteristics of the study participants were presented according to the AMH quintile category. The primary outcome was incident VMS before menopause. Each participant was followed up from the baseline examination until the time of VMS occurrence, time of menopause, or last questionnaire survey date (October 4, 2020), whichever came first. New-onset VMS was assessed based on the MENQoL^25,26^, wherein the time frame is “in the Past month.” Likewise, VMS was known to have occurred between the two visits (visit with first report of VMS and the previous visit) but the exact time when it developed was unknown; thus, a parametric proportional hazards model was used to account for this type of interval censoring (stpm command in Stata)^27^ and to estimate adjusted hazard ratios (HRs) and 95% CIs for new-onset VMS according to AMH level. In these models, the baseline hazard function was parameterized with restricted cubic splines in log time with four degrees of freedom.

The statistical models were adjusted for age, educational attainment, parity (nulliparous or parous), physical activity, smoking status, alcohol intake, medication use for hyperlipidemia, hypertension, and BMI. We conducted subgroup analyses by age (< median vs. ≥ median), and BMI (< 23 kg/m^2^ vs. ≥ 23 kg/m^2^, cutoff value for overweight in Asian population^20^). We used likelihood ratio tests comparing models with and without multiplicative interaction terms to evaluate the interactions. Statistical analyses were performed using STATA version 16.0 (Stata Corp LP; College Station, TX, USA). Two-sided *P* values < 0.05 were considered as statically significant.

### Ethical approval

The present study does not include any animal studies. All procedures involved in this study of human participants were in accordance with the ethical standards of the institutional research committee and with the 1964 Helsinki declaration and its later amendments or comparable ethical standards. Written informed consent was obtained from all participants included in this study. This cohort study was approved by the Institutional Review Board of Kangbuk Samsung Hospital (2021-02-023).

## Results

### Baseline characteristics

The baseline characteristics of the study participants are shown in Table 1. At baseline, the mean age of women exclusively in the premenopausal stage was 44.74 years (SD, 2.38 years) and the median AMH level was 0.69 ng/mL (interquartile range, 0.26–1.35 ng/mL). AMH levels were inversely associated with age, hypertension, medication use for hyperlipidemia, obesity, and BMI (Table 1).

**Table 1:** Baseline characteristics of premenopausal Korean women free of vasomotor symptoms (n = 2,041).

Characteristics	Overall	Quintiles AMH^a^ level (range), ng/ml					*P* for trend
		Q1 (0.01–0.164)	Q2 (0.165–0.433)	Q3 (0.437–0.837)	Q4 (0.838–1.55)	Q5 (1.56–30.99)	
Age(years)^b^	44.74 ± 2.38	46.24 ± 2.80	45.47 ± 2.38	44.51 ± 2.04	43.97 ± 1.83	43.50 ± 1.56	< 0.001
Age at menarche (years) ^b^	13.92 ± 1.38	13.98 ± 1.42	13.99 ± 1.31	13.88 ± 1.37	13.87 ± 1.38	13.90 ± 1.42	0.180
Parity^c^(%)	92.52	90.82	94.33	92.51	93.32	91.64	0.868
Ever smoker(%)	10.62	8.85	9.75	11.88	10.44	12.22	0.126
Alcohol intake(%)^d^	10.73	7.40	11.90	8.65	14.50	11.14	0.040
HEPA (%)^e^	15.49	17.52	17.33	15.92	14.84	11.78	0.015
High education(%)^f^	81.05	78.66	79.80	79.29	81.08	86.43	0.007
Diabetes (%)	1.72	1.71	2.70	1.48	1.21	1.50	0.350
Hypertension (%)	3.98	7.09	3.93	3.20	3.39	2.26	0.001
Lipid lowering agent (%)	1.57	2.68	1.72	1.72	0.97	0.75	0.020
Obesity (%)	15.73	18.98	16.95	14.95	16.22	11.44	0.006
Body mass index, kg/m^2^	22.31 ± 2.92	22.71 ± 3.11	22.57 ± 3.11	22.10 ± 2.82	22.27 ± 2.84	21.87 ± 2.60	< 0.001

^a^AMH = Anti-Müllerian Hormone, Data are ^b^ mean ± standard deviation, ^c^Parity including live births and stillbirths, ^d^ ≥ 10 g of ethanol per day, ^e^HEPA = health-enhancing physical activity, ^f^ College graduate or higher.

### AMH levels and incident VMS

**Figure 2:**
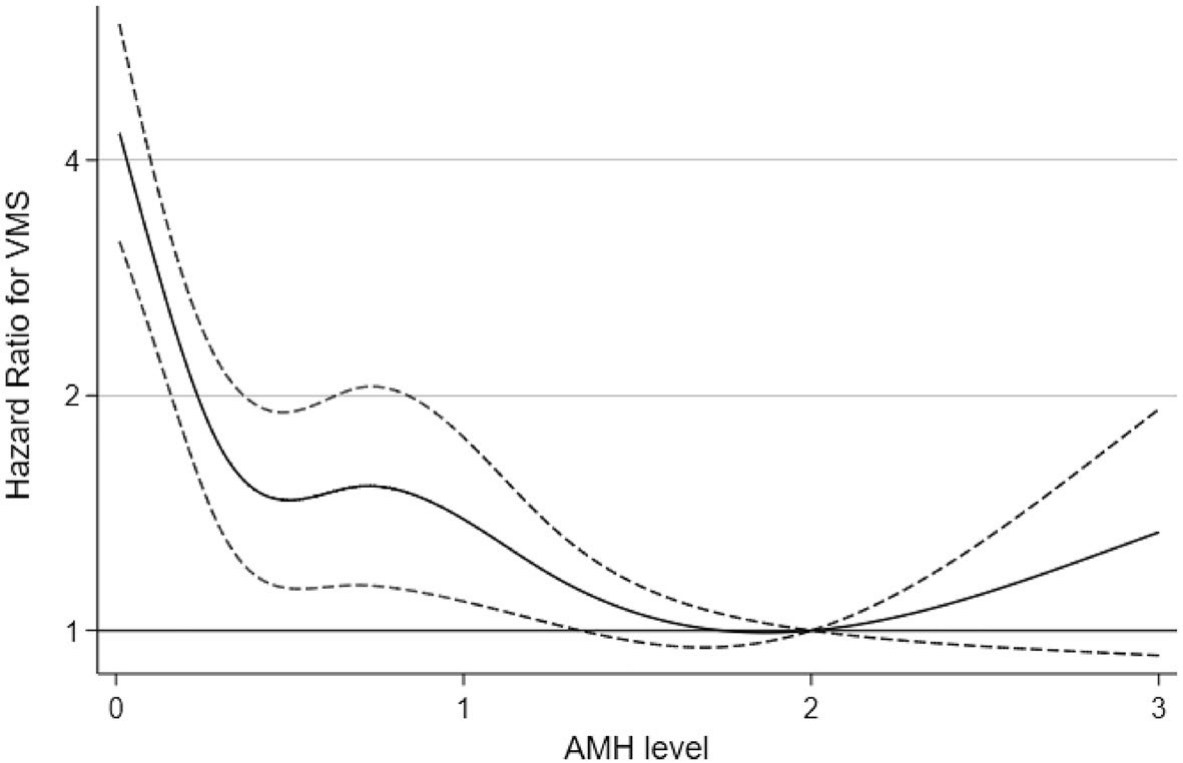
Multivariate-adjusted hazard ratios (95% confidence intervals) for incident early-onset vasomotor symptoms using AMH levels as a continuous variable. The curves represent adjusted hazard ratios (solid line) and their 95% confidence intervals (dashed lines) for incident early-onset VMS on the basis of restricted cubic splines for AMH levels with knots at the 5th, 27.5th, 50th, 72.5th, and 95th percentiles. The model was adjusted for age, education level, parity, physical activity, smoking status, alcohol intake, hypertension, lipid lowering agent and body mass index.

During a median follow-up of 4.4 years (interquartile range, 3.9–5.3 years), a total of 708 women developed incident VMS before menopause. The median frequency of visits was 3 visits (interquartile range: 2–3 visits). The menopausal stage at the time of first report for VMS during the follow-up was premenopausal stage for 306 (43.2%), early menopausal transition for 98 (13.8%) and late menopausal transition for 304 (42.9%) among participants who developed new-onset of VMS (n = 708). In the spline regression models to assess the relationship between the AMH level as a continuous variable and the development of VMS, the incidence of VMS decreased as the AMH levels increased (Fig. 2). The AMH quintiles were statistically significantly and inversely associated with the risk of incident VMS (Table 2). After adjustment for age, education level, parity, physical activity, smoking status, alcohol intake, medication use of hyperlipidemia, hypertension, and BMI, multivariable-adjusted HR (95% CIs) for incident VMS comparing AMH quintiles 4–1 to the highest quintile were 1.02 (0.78–1.33), 1.37 (1.06–1.76), 1.36 (1.04–1.76), and 2.38 (1.84–3.08), respectively (P for trend < 0.001) (Table 2). The AMH level as a continuous variable was inversely associated with the incident VMS with a multivariable-adjusted HR of 1.28 (95% CI, 1.16–1.41) per 1-ng/mL decrease in the AMH level. When BMI category was considered a categorical variable in the model where BMI was categorized according to the Asian-specific criteria^20^ as underweight (BMI < 18.5 kg/m^2^), normal weight (BMI 18.5–22.9 kg/m^2^), overweight (BMI 23–24.9 kg/m^2^), and obese (BMI ≥ 25 kg/m^2^), and the findings were consistent with the original findings (Table S1).

**Table 2:** Incidence of early-onset vasomotor symptoms by AMH quintiles in premenopausal women (n = 2041).

AMH level	Person-years (PY)	No of incident early-onset VMS	Incidence rate (cases per 100 PY)	Age-adjusted HR (95% CI)	Multivariable-adjusted HR (95% CI)
Quintile 5	1862.5	105	5.6	Reference	Reference
Quintile 4	1911.8	118	6.2	1.06 (0.82–1.39)	1.02(0.78–1.33)
Quintile 3	1845.8	145	7.9	1.40 (1.08–1.80)	1.37(1.06–1.76)
Quintile 2	1729.0	145	8.4	1.41(1.09–1.83)	1.36(1.04–1.76)
Quintile 1	1467.1	195	13.3	2.43(1.88–3.13)	2.38(1.84–3.08)
*P* for trend				< .001	< .001
AMH as a continuous variable	8816.2	708	8.0	1.29 (1.17–1.42)	1.28 (1.16–1.41)

Parametric proportional hazard models were used to estimate the hazard ratios with 95% confidence intervals. The multivariable model was adjusted for age, education level, parity, physical activity, smoking status, alcohol intake, hypertension, lipid lowering agent and body mass index.

Abbreviation: AMH, anti-Müllerian hormone; CI, confidence interval; HR, hazard ratio.

When we compared the characteristics of participants with successful follow-up with information on VMS and those lost to follow-up, those lost to follow-up were more likely to be older (Table S2). In analyses using inverse probability weights based on the determinants of having a follow-up visit over the study period to account for the possibility of selection bias, the results were very similar to the original results (Table S3).

### Subgroup analyses

The inverse association between the AMH level and incident VMS was consistently observed in subgroups without statistically significant interaction by age groups and the presence of overweight (Table 3). Multivariable-adjusted HR (95% CI) for incident VMS comparing the lowest AMH quintile to the highest quintile was 2.60 (1.82–3.72) in younger women (below the median age) and 2.30 (1.54–3.45) in older women, while corresponding HR (95% CI) was 2.70 (1.94–3.75) in non-overweight women and 2.05 (1.35–3.11) in overweight women (Table 3).

**Table 3:** Incident early-onset vasomotor symptoms according to AMH quintiles in clinically relevant subgroups.

AMH level	Multivariable-adjusted HR (95% CI)			
	Age		BMI	
	< median ^a^ N = 1,021	≥ median ^a^ N = 1,020	< 23 kg/m^2^ N = 1, 355	≥ 23 kg/m^2^ N = 686
Quintile 5	Reference	Reference	Reference	Reference
Quintile 4	1.04(0.75–1.45)	0.95(0.60–1.49)	1.20 (0.85–1.68)	0.77 (0.50–1.19)
Quintile 3	1.21(0.86–1.72)	1.51(1.01–2.28)	1.43 (1.03–1.98)	1.31 (0.86–2.00)
Quintile 2	1.05(0.70–1.58)	1.52(1.02–2.27)	1.30 (0.92–1.85)	1.44 (0.96–2.16)
Quintile 1	2.60(1.82–3.72)	2.30(1.54–3.45)	2.70 (1.94–3.75)	2.05 (1.35–3.11)
*P* for trend	< 0.001	< 0.001	< 0.001	< 0.001
*P* for interaction	0.149		0.280	

^a^ Median of age—44.27.

Parametric proportional hazard models were used to estimate the hazard ratios with 95% confidence intervals. The multivariable model was adjusted for age, menopause stage, menarche age, education level, parity, physical activity, smoking status, body mass index, and alcohol intake. The likelihood ratio tests comparing models with and without multiplicative interaction terms to evaluate the interactions.

Abbreviation: AMH, anti-Müllerian hormone; CI, confidence interval; HR, hazard ratio.

## Discussion

In the present cohort study of middle-aged Korean women, we found an inverse association between AMH levels and the risk of incident VMS in premenopausal women and this association remained statistically significant after adjusting for age and other potential confounders.

Evidence suggests that early-onset VMS most likely represents a physiological response to the hormonal fluctuations during menopausal transition^8^. However, findings on the relationship between reproductive hormones and VMS have been inconsistent. Despite the established role of estrogen in VMS owing to its role in thermoregulation^10–12^, the urinary, plasma, or vaginal levels of estrogen are not consistently associated with VMS^9,13^. AMH is considered a reliable marker of ovarian function and a predictor of the time to menopause, along with the strength of its stability during the menstrual period^14–16^. Until now, studies on the effect of AMH on VSM risk in comparison with other reproductive hormones are very limited. A recent cross-sectional study of 108 women aged 40–59 years by Dhanoya et al.^13^ demonstrated for the first time that AMH and FSH, but not estradiol, were statistically significantly associated with hot flashes^13^. However, no study has specifically explored the longitudinal relationship between AMH and VMS. In our study of exclusively premenopausal stage women, we observed a statistically significant association between AMH levels and the development of VMS, even after controlling for age, suggesting that AMH may independently predict the incidence of VMS. We also focused on early-onset VMS occurring before menopause as previous studies applied^8^. Considering that distinct mechanisms may play a role in early onset VMS and late-onset VMS^8^, it may be worth investigating whether AMH is differentially associated with early-onset and late-onset VMS across the menopausal transition. Whether AMH has a causal role in the development of VMS or merely reflects the progression through menopausal transition is unclear. In our study, the relationship between AMH levels and VMS risk could be explained based on the predictive role of AMH for progression to menopausal transition^10,13^, given that recent studies have reported that AMH levels are better predictive indicators when a final menstrual period may occur later among late reproductive-age women than FSH and other reproductive hormones^15,16^. In the study by Dhanoya et al.^13^ the association between menopausal status and hot flushes was no longer statistically significant when AMH was considered, suggesting that AMH is a predictive marker of transition through menopause, minimizing the misclassification of menopausal stage^13^. While our study has the advantage of a longitudinal design and relatively young women in exclusively premenopausal stage, we did not measure estrogen or FSH levels, which is a limitation of our study. Future studies should explore the role of AMH in the occurrence of VMS with respect to other reproductive hormones to confirm our findings.

Our study had some limitations. First, AMH was measured only at baseline; thus, we could not consider changes in AMH levels during the follow-up. Second, besides AMH, data on other reproductive hormones were not available, limiting our ability to evaluate the role of AMH in comparison with other hormonal levels. Female sex hormone levels typically vary throughout the menstrual cycle, whereas AMH level remain constant during the menstrual cycle; this stability relative to other female hormones is one of the strengths of our study^24^. Because of the variability in reproductive hormone levels throughout the menstrual cycle, the hormone levels need to be measured at the right time in the early follicular phase, which typically overlaps with the menstruation period. However, our study participants were enrolled during comprehensive screening examinations, which include urinalysis and pap smears as a basic test that women were instructed to avoid during menstruation corresponding to the early follicular phase. Thus, accurate measurements of serum reproductive hormone levels were not feasible owing to the nature of our health checkup examination^19^. Third, although the incident VMS in our study occurred before the menopause, the detailed information on the onset of VMS and VMS duration was not available. Among 708 incident VMS cases, only 70 reached menopause until the end of the study period and the interval between the first report of VMS and menopause was a median of 2.0 years (interquartile range, 1.3–3.2 years, maximum 5 years). Since the enrolment of this cohort ended in 2018, only a small proportion of participants experienced menopause, thus limiting further detailed analyses incorporating the duration of VMS and the interval between the onset of VMS and menopause. Furthermore, new-onset VMS was assessed based on the MENQoL, wherein the time frame is “in the Past month.” This limits the exact estimations of VMS onset time and duration since the study participants visited the health checkup centers every 1–2 years or completed the MENQoL questionnaire at the time when their menopausal stage progressed to the subsequent stage. Fourth, confounders such as smoking status, alcohol intake, and physical activity were assessed using self-reported questionnaires, which may have led to misclassification. Additionally, we could not exclude unmeasured confounders. Fifth, our study findings in middle-aged Korean women may not be readily generalizable to other populations with different race/ethnicity compositions. The strengths of our study include its prospective design; the large sample size of a well-characterized population of premenopausal women; and the use of carefully standardized clinical, lifestyle, and laboratory measures, which allowed us to account for multiple potential confounders.

In this cohort study of middle-aged Korean women whose menopausal status was exclusively in the late reproductive stage, AMH levels were inversely associated with the risk of incident VMS before menopause. Our results support that AMH levels indicative of ovarian reserve function may help identify premenopausal stage women at a high risk of early-onset VMS. Further studies should confirm our findings in different populations and elucidate the role of AMH and other reproductive hormones in the pathogenesis of early-onset VMS.

## Supplementary Information

Supplementary Information.

## Data Availability

Some or all datasets generated during and/or analyzed during the current study are not publicly available. However, analytical methods and study materials are available from the corresponding author on reasonable request.

## References

[1] Thurston RC, Joffe H (2011). Vasomotor symptoms and menopause: findings from the Study of Women's Health across the Nation. Obstet. Gynecol. Clin. North Am..

[2] Pinkerton JV, Abraham L, Bushmakin AG, Cappelleri JC, Komm BS (2016). Relationship between changes in vasomotor symptoms and changes in menopause-specific quality of life and sleep parameters. Menopause.

[3] Avis NE (2015). Duration of menopausal vasomotor symptoms over the menopause transition. JAMA Intern. Med..

[4] Freeman EW, Sammel MD, Lin H, Liu Z, Gracia CR (2011). Duration of menopausal hot flushes and associated risk factors. Obstet. Gynecol..

[5] Freeman EW, Sammel MD, Sanders RJ (2014). Risk of long-term hot flashes after natural menopause: evidence from the Penn Ovarian Aging Study cohort. Menopause.

[6] Tepper PG (2016). Characterizing the trajectories of vasomotor symptoms across the menopausal transition. Menopause.

[7] Thurston RC (2016). Trajectories of vasomotor symptoms and carotid intima media thickness in the Study of Women's Health Across the Nation. Stroke.

[8] Zhu D (2020). Vasomotor menopausal symptoms and risk of cardiovascular disease: A pooled analysis of six prospective studies. Am. J. Obstet. Gynecol..

[9] Freedman RR (2014). Menopausal hot flashes: mechanisms, endocrinology, treatment. J. Steroid Biochem. Mol. Biol..

[10] Randolph JF (2005). The relationship of longitudinal change in reproductive hormones and vasomotor symptoms during the menopausal transition. J. Clin. Endocrinol. Metab..

[11] Gold EB (2007). Relation of daily urinary hormone patterns to vasomotor symptoms in a racially/ethnically diverse sample of midlife women: Study of women's health across the nation. Reprod. Sci..

[12] Freedman RR (1998). Biochemical, metabolic, and vascular mechanisms in menopausal hot flashes. Fertil. Steril..

[13] Dhanoya T (2016). Hot flushes and reproductive hormone levels during the menopausal transition. Maturitas.

[14] La Marca A (2010). Anti-Mullerian hormone (AMH) as a predictive marker in assisted reproductive technology (ART). Hum. Reprod. Update.

[15] Freeman EW, Sammel MD, Lin H, Gracia CR (2012). Anti-mullerian hormone as a predictor of time to menopause in late reproductive age women. J. Clin. Endocrinol. Metab..

[16] Finkelstein JS (2020). Antimullerian hormone and impending menopause in late reproductive age: The Study of Women's Health Across the Nation. J. Clin. Endocrinol. Metab..

[17] Craig CL (2003). International physical activity questionnaire: 12-country reliability and validity. Med. Sci. Sports Exerc..

[18] Harlow SD (2012). Executive summary of the Stages of Reproductive Aging Workshop + 10: addressing the unfinished agenda of staging reproductive aging. J. Clin. Endocrinol. Metab..

[19] Kim Y (2022). Menopausal stages and prevalence of thyroid dysfunction. Thyroid.

[20] World Health Organization & Regional Office for the Western Pacific. *The Asia-Pacific perspective: redefining obesity and its treatment*. (Health Communications Australia, 2000).

[21] Whelton PK (2018). 2017 ACC/AHA/AAPA/ABC/ACPM/AGS/APhA/ASH/ASPC/NMA/PCNA guideline for the prevention, detection, evaluation, and management of high blood pressure in adults: A report of the American College of Cardiology/American Heart Association Task Force on Clinical Practice Guidelines. J. Am. Coll. Cardiol..

[22] Clinical and Laboratory Standards Institute. *EP15-A2: User Verification of Performance for Precision and Trueness; Approved guideline*. 2nd edn, (Clinical and Laboratory Standards Institute, 2012).

[23] Clinical and Laboratory Standards Institute. *EP17-A2: Evaluation of detection capability for clinical laboratory measurement procedures; Approved guideline*. 2nd edn, (Clinical and Laboratory Standards Institute, 2012).

[24] Streuli I (2008). Serum antimüllerian hormone levels remain stable throughout the menstrual cycle and after oral or vaginal administration of synthetic sex steroids. Fertil. Steril..

[25] Hilditch JR (1996). A menopause-specific quality of life questionnaire: Development and psychometric properties. Maturitas.

[26] Park JH, Bae SH, Jung YM (2020). Validity and reliability of the Korean version of the menopause-specific Quality of Life. J. Korean Acad. Nurs..

[27] Royston P, Parmar MK (2002). Flexible parametric proportional-hazards and proportional-odds models for censored survival data, with application to prognostic modelling and estimation of treatment effects. Stat Med.

