# Supplementary material for: Low anti-Müllerian hormone levels are associated with an increased risk of incident early-onset vasomotor symptoms among premenopausal women
Source: Sci Rep. 2022 Jul 13;12:11904. doi: 10.1038/s41598-022-16182-7 (PMC9279494; doi:10.1038/s41598-022-16182-7)
Supplement: Supplementary file 1 — Supplementary Information. [file 41598_2022_16182_MOESM1_ESM.docx]

**Supplementary data**

**Low anti-Mullerian hormone levels are associated with an increased risk of incident early-onset vasomotor symptoms among premenopausal women**

SunJu NamGoung*, Yoosoo Chang*, Yejin Kim, Hoon Kim, In Young Cho, Ria Kwon, Gayoung Lim, Hye Rin Choi, Jeonggyu Kang, Kye-Hyun Kim, Yun Soo Hong, Di Zhao, Hyun-Young Park, Juhee Cho, Eliseo Guallar, Min-Jung Kwon**, Seungho Ryu**

**Table S1.** Longitudinal association between anti-Mullerian hormone levels and VMS incidence among premenopausal women free of VMS at the baseline based on parametric proportional hazard models including BMI as a categorical variable (BMI <18.5, 18.5–22.9, 23–24.9 and ≥25 kg/m^2^) instead of BMI as a continuous variable.

| AMH level | Multivariate-adjusted HR (95% CI)^a^ |
| --- | --- |
| Quintile 5 | Reference |
| Quintile 4 | 1.02 (0.78-1.34) |
| Quintile 3 | 1.38 (1.07-1.79) |
| Quintile 2 | 1.38 (1.07-1.80) |
| Quintile 1 | 2.43 (1.88-3.15) |
| *P* for trend | <.001 |
| AMH as a continuous variable | 1.29 (1.16-1.42) |

^a^ Parametric proportional hazard models were used. The multivariate model was adjusted for age, education level, parity, physical activity, smoking status, alcohol intake, hypertension, lipid lowering agent and body mass index category. BMI was categorized as underweight (BMI <18.5 kg/m^2^), normal weight (BMI 18.5–22.9 kg/m^2^), overweight (BMI 23–24.9 kg/m^2^) and obesity (BMI ≥25 kg/m^2^).

Abbreviations: *BMI*, body mass index; *CI*, confidence interval; *HR*, hazard ratio; *VMS*, vasomotor symptoms.

**Table S2.** Baseline characteristics of the study participants according to follow-up

| Characteristics | No follow up  (n = 1011) | Follow up  (n = 2041) |
| --- | --- | --- |
| Age (y) | 45.18±2.57 | 44.74±2.38 |
| AMH (ng/mL) ^a^ | 0.69 (0.26-1.35) | 0.56 (0.18-1.21) |
| Age at menarche (years) ^b^ | 14.12±1.44 | 13.92±1.38 |
| Parity^c^(%) | 91.93 | 92.52 |
| Ever smoker (%) | 12.27 | 10.62 |
| Alcohol intake (%)^d^ | 15.41 | 10.73 |
| HEPA (%) | 15.93 | 15.49 |
| High education (%)^e^ | 78.06 | 81.05 |
| Diabetes (%) | 1.98 | 1.72 |
| Hypertension (%) | 5.77 | 3.98 |
| Lipid lowering agent (%) | 1.78 | 1.57 |
| Obesity (%) | 15.13 | 15.73 |
| Body mass index, kg/m^2^ | 22.42±2.94 | 22.31±2.92 |

Data are ^a^ median (interquartile range), ^b^ mean±standard deviation, or percentage

^c^Parity indicative of live births and stillbirths

^d^≥10 g of ethanol per day

^e^ College graduate or higher

Abbreviations: AMH, anti-Mullerian hormone; HEPA, health-enhancing physical activity

**Table S3.** Longitudinal association between anti-Mullerian hormone levels and incident VMS among premenopausal women free of VMS at the baseline based on parametric proportional hazard models using inverse probability weights

| AMH level | Multivariate-adjusted  HR (95% CI)^a^ |
| --- | --- |
| Quintile 5 | Reference |
| Quintile 4 | 0.98 (0.74-1.30) |
| Quintile 3 | 1.33 (1.02-1.73) |
| Quintile 2 | 1.23 (0.93-1.63) |
| Quintile 1 | 2.27 (1.73-2.99) |
| *P* for trend | <.001 |
| AMH as a continuous variable | 1.23 (1.09-1.38) |

^a^ Parametric proportional hazard models using inverse probability weights were used. The multivariate model was adjusted for age, education level, parity, physical activity, smoking status, alcohol intake, hypertension, lipid lowering agent and body mass index.

Abbreviations:  *CI*, confidence interval; *HR*, hazard ratio; *VMS*, vasomotor symptoms.
